# High-Throughput Cardiac Hypertrophy Phenotyping Supports Lead Optimization of GRK5 Inhibitors

**DOI:** 10.1021/acsmedchemlett.5c00528

**Published:** 2025-12-15

**Authors:** Pia Steinkuhl, Anca Kliesow Remes, Carmen Carrillo García, Amol Sonawane, Ranjith Kumar Gadi, Arun K. Ghosh, John J. G. Tesmer, Oliver J. Müller, Dennis Schade

**Affiliations:** † Department of Pharmaceutical & Medicinal Chemistry, 9179 Christian-Albrechts-University of Kiel, Kiel 24118, Germany; ‡ Department of Internal Medicine V, 15056 University Medical Center Schleswig-Holstein, Campus Kiel, Kiel 24105, Germany; § German Center for Cardiovascular Research, (DZHK), partner site Hamburg/Kiel/Lübeck, Kiel 24105, Germany; ∥ Department of Chemistry, 311308 Purdue University, West Lafayette, Indiana 47907, United States; ⊥ Department of Medicinal Chemistry and Molecular Pharmacology, 311308 Purdue University, West Lafayette, Indiana 47907, United States; # Department of Biological Sciences, 311308 Purdue University, West Lafayette, Indiana 47907, United States

**Keywords:** Morphological profiling, high-content imaging, coculture assay, disease modeling, GPCR signaling, structure−activity relationship, drug development

## Abstract

Cardiac hypertrophy poses a clinical challenge in heart failure progression with limited therapeutic options to reverse the process of pathological remodeling. We present a high-throughput phenotypic screening assay designed to support lead optimization from novel approaches. A mixed cell culture from neonatal rat hearts was established, allowing simultaneous assessment of cardiomyocytes and noncardiomyocytes within a shared physiologically relevant microenvironment. Customized CellProfiler-based image analysis extracted multiparametric morphological data that was merged in a “Hypertrophy Score” metric for quantitative analyses. The assay was applied to investigate G protein-coupled receptor kinase 5 (GRK5) as a promising target in cardiac hypertrophy. Structure–activity and −property relationships from a focused GRK5 inhibitor collection revealed a negative influence on cellular efficacy by lipophilic and covalently reactive compounds. Correlating biochemical with cellular data eliminated GRK6 as a common off-target concern and underlined the value of potent GRK5 versus GRK2 inhibition. Diastereomers **4a/b** were identified as valuable chemical probes.

## Graphical abstract

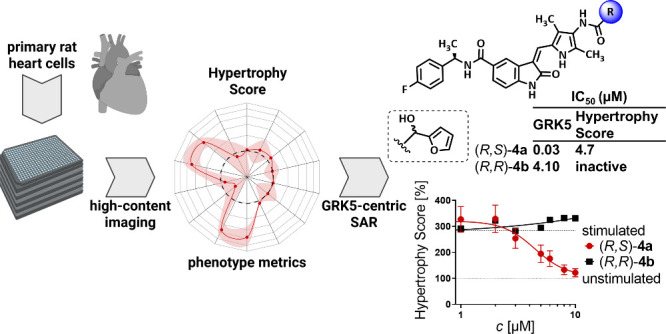

Cardiovascular diseases (CVDs) remain a leading cause of morbidity and mortality worldwide.^1^ In many CVDs, cardiac hypertrophy is a key pathological adaptation, initially compensating for increased workload but ultimately leading to irreversible remodeling, dysfunction, and heart failure.^2,3^ In addition to biomechanical stress, several neurohumoral systems contribute to the development of hypertrophy,^4,5^ including the sympathetic nervous system,^6^ the renin-angiotensin-aldosterone system (RAAS),^7^ cytokines and inflammatory mediators.^8,9^ At the cellular level, hypertrophy is characterized by increased cardiomyocyte (CM) size, elevated protein synthesis and a regression to the fetal gene program,^10^ with increased expression levels of hypertrophic markers like the cardioprotective hormones atrial natriuretic peptide (ANP) and B-type natriuretic peptide (BNP).^2,3^ Other cardiac cell types also contribute to hypertrophic development, including cardiac fibroblasts promoting fibrosis by increased proliferation and extracellular matrix production.^11^ In contrast, crosstalk between endothelial cells and CMs induces the expression of protective factors and cytokines by both cell types.^12^

For lead optimization campaigns, *in vivo* models offer physiological relevance; however, they are impractical for high-throughput screening (HTS) approaches.^13,14^ Therefore, cell cultures derived from neonatal rat hearts are widely used, maintaining key physiological features such as contractility and supporting HTS for drug discovery.^15^ Neonatal rat cardiomyocytes (NRCMs) usually are purified from other cardiac cell types, enabling a targeted analysis of their biology.^16^ However, this approach does not fully capture the complex composure of the *in vivo* heart, missing crucial aspects of intercellular communication.^17^ To enhance the *in vivo* translation of this model and increase the likelihood of identifying effective therapies, further advancements in physiological relevance, readouts and high-throughput capabilities are essential.^18^ It is crucial to select appropriate *in vitro* models that replicate the pathophysiological disease features while remaining cost-effective and balancing simplicity with complexity to generate meaningful insights. Additionally, relying solely on manual CM size measurements provides only an incomplete and potentially biased assessment of drug effects. By leveraging modern computational tools, more complex data can be extracted from cell images, enabling a deeper understanding of phenotypic changes. Furthermore, enhancing the throughput capacity of the model will enable the screening of a larger number of potential drug candidates. This ultimately increases the chances of identifying effective treatments for cardiac hypertrophy.

Here, we developed a high-throughput assay for the phenotypic characterization of cardiac hypertrophy under pathophysiological conditions, aiming to accelerate the translation of early lead candidates into *in vivo* disease models. The system was used to evaluate GRK5 as a promising therapeutic target via structure–activity relationship (SAR) studies of a sunitinib-derived inhibitor collection. This approach identified compound **4a** as a viable early lead with both antihypertrophic and antiproliferative properties, indicating a potential dual mechanism of action.

## Semiautomated Image Analysis Pipeline for the Mixed Cardiac Cell Culture

To increase the robustness and information quantity of the phenotypic readout, we first established a high-content, semiautomated image analysis pipeline for cardiac hypertrophy (Figure 1A). We selected the CellProfiler software because its open-source nature allows broad applicability across research laboratories, regardless of the available microscopy systems or computational infrastructure, offering a highly accessible solution for diverse experimental setups.^19,20^

**Figure 1:**
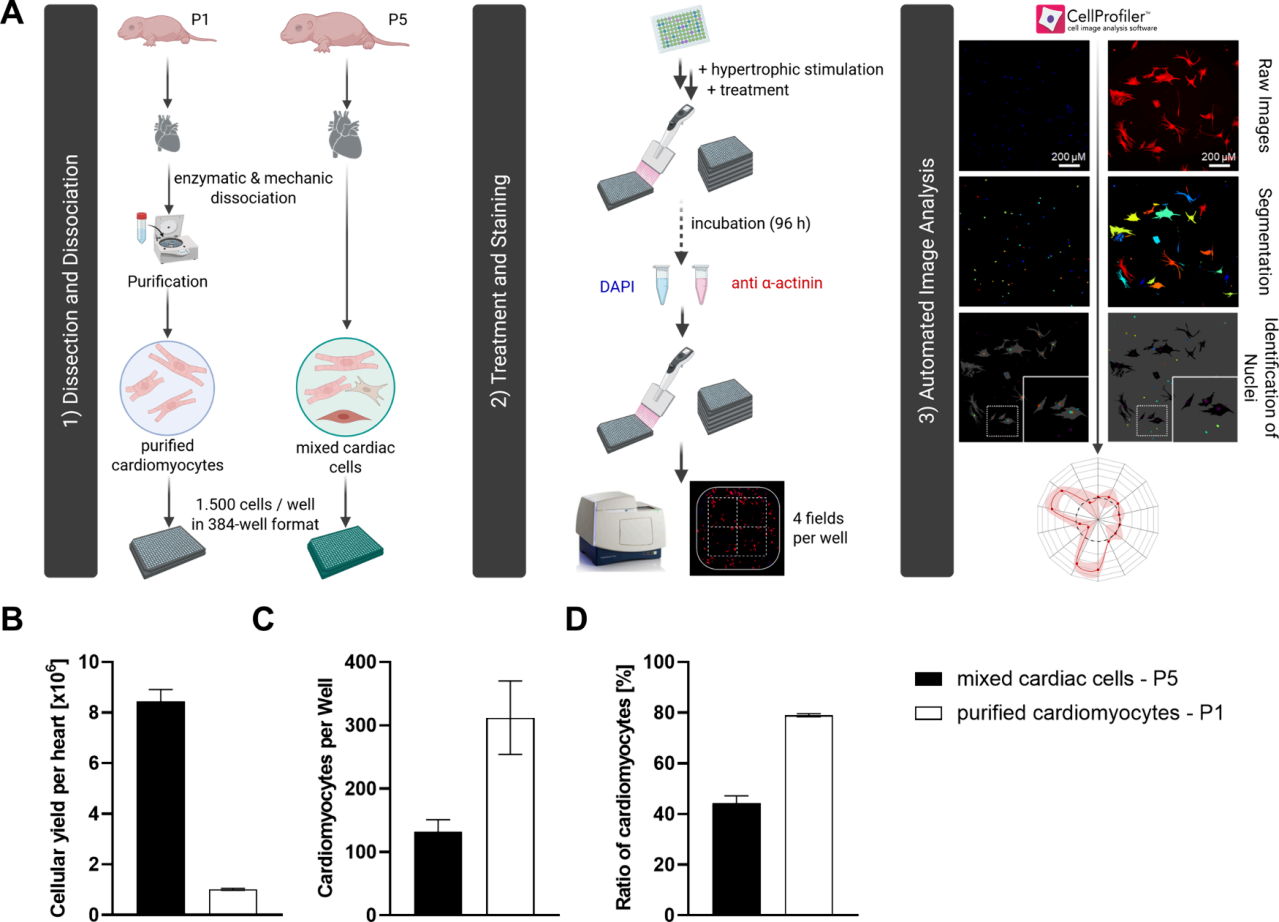
Comparison of high-throughput and high-content assay platforms for hypertrophic phenotype assessment in mixed cardiac cells versus purified CMs. (A) Neonatal rat hearts are either purified via Percoll gradient centrifugation to isolate CMs, or used as a mixed cardiac cell population retaining all cardiac cell types. Cells are seeded into 384-well plates and stimulated with pro-hypertrophic agents, followed by treatment with test compounds after 24 h. After 96 h, cells are fixed and stained for α-actinin and nuclei. Imaging (4 fields/well) and automated high-content analysis with a customized CellProfiler pipeline enables quantitative evaluation of CM hypertrophy and cell viability. (B) Mixed cardiac cells from 5-day-old-rats (P5) results in higher cell yield compared to P1-derived cells. (C) Semiautomated image analysis enables robust CM quantifications in both P1- and P5-derived heart cell preparations. (D) Mixed cardiac cells maintain a high proportion of CMs without fibroblast overgrowth, preserving a balanced, more physiological environment. Data represents mean ± SEM (*n* ≥ 5).

All cells were stained with DAPI, and CM were specifically labeled with an anti-α-actinin antibody for morphological analysis. In the first step, the illumination of α-actinin stained images was corrected, thereby enhancing the signal-to-noise ratio and reducing background. This preprocessing step facilitated accurate segmentation of the heterogeneous cardiomyocyte morphology, overcoming the limitations of standard propagation-based methods that often misclassified binucleated cardiomyocytes. Next, CM and non-CM nuclei were distinguished by colocalizing DAPI and α-actinin signals, enabling a more precise evaluation of the heterogeneous cell culture model. Because CMs frequently exhibit binucleation as part of their growth process, nuclearity (i.e., number of nuclei per CM) was included as a parameter. The “RelateObjects” module was used to assign all nuclei within a single CM as child objects of that cell. This also enabled the creation of subpopulations, such as mononucleated cells. With these nuclei and CM populations, various measurements were performed for morphological evaluation. This included quantitative measurements, nuclear and cellular area, cellular intensity, and texture, broadening the spectrum of phenotypic characterization beyond mere cellular area measurement (see Supporting Information).

To assess the advantages and limitations of our mixed cardiac cell approach, the protocol was compared to an established procedure that employs CMs enriched through purification.^21^ A key distinction between the two protocols was the age of the neonatal rats: our mixed cardiac cell culture is derived from five-day-old (P5) rats, whereas the CM purification protocol uses one-day-old (P1) rats. P1 CMs have a more immature protein expression profile and a lower tendency for binucleation, potentially influencing hypertrophic responses. Altering the cellular composition affects the baseline characteristics of the assay. P5 rats yielded 8.4 × 10^6^ cells per heart after dissociation, whereas the smaller, less developed P1 hearts yielded only 1.0 × 10^6^ purified cells per heart (Figure 1B). From a high-throughput perspective, P5-derived cultures are advantageous as they generate a higher cellular yield per animal, reducing the number of animals required for experiments. However, the purification step increases the ratio of CM in culture. At a seeding density of 1,500 cells per well, the mixed cardiac cell culture contained approximately 132 CMs per well, compared to 312 in the purified culture (Figure 1C), corresponding to a CM ratio of 44% versus 79%, respectively (Figure 1D). This near doubling of CMs per well with purification enhances the statistical power of the analysis, as mean values are calculated from a larger cell population.

## Phenotypic Assessment of Selected Hypertrophy Stimulants

The new CellProfiler pipeline was employed for systematic profiling of a selection of well-characterized hypertrophic stimulants^22^ targeting different pathophysiologically relevant signaling pathways. This set included the adrenergic agonists phenylephrine (PE) and norepinephrine (NE), given that excessive adrenergic stimulation is a key driver of pathological cardiac hypertrophy,^6^ and the endogenous peptides endothelin-1 (ET-1) and angiotensin-II (Ang. II), both of which are elevated in patients with cardiac hypertrophy and commonly used for *in vitro* stimulation.^22^ Also included were the cytokines leukemia inhibitory factor (LIF)^23,24^ and transforming growth factor beta (TGFβ), along with cortisol. Each stimulant was applied at three different concentrations, except for timolol (TML), which was tested as a β-antagonist in combination with PE to evaluate whether α-adrenergic stimulation remains dominant when β-adrenergic signaling is blocked.^22^ LIF was also tested individually and in combination with PE, to have a bifunctional stimulation representing multiple relevant pathways (see Table S1 of Supporting Information for a detailed overview of compounds and treatment conditions).

PE-LIF induced significant changes in key morphological features (Figure 2B), most notably an increase in CM area compared to DMSO vehicle control. Radar plots of all hypertrophy phenotypes are shown in Supporting Information, Figure S1. Because α-actinin is a key structural protein of the sarcomere, its staining intensity and distribution reflect changes in sarcomere organization during hypertrophy. As expected, hypertrophic remodeling led to alterations in α-actinin staining intensity and texture measurements, suggesting structural reorganization and disruptions in sarcomere composition. Only a slight yet consistent increase in CM nuclearity was detected. Interestingly, the response patterns of mononucleated CMs closely mirrored those of the total CM population (including both mono- and binucleated cells), indicating that nuclearity did not provide additional discriminatory power in assessing hypertrophic stimulation in this context. Although increased nuclear area in hypertrophic CMs has been reported,^25^ this feature is rarely used *in vitro* and has (so far) not been demonstrated in mixed cardiac cell cultures. Here, nuclear enlargement was observed specifically in CMs but not non-CMs. To assess potential cardiotoxicity, total nuclear counts, along with CM and non-CM subpopulations, were analyzed alongside CM numbers. As expected, none of the hypertrophic stimulants led to a significant reduction in cell populations. This aligns with previous reports, as all tested stimulants are physiological substances known to induce hypertrophy rather than exert toxic effects.^22^

**Figure 2:**
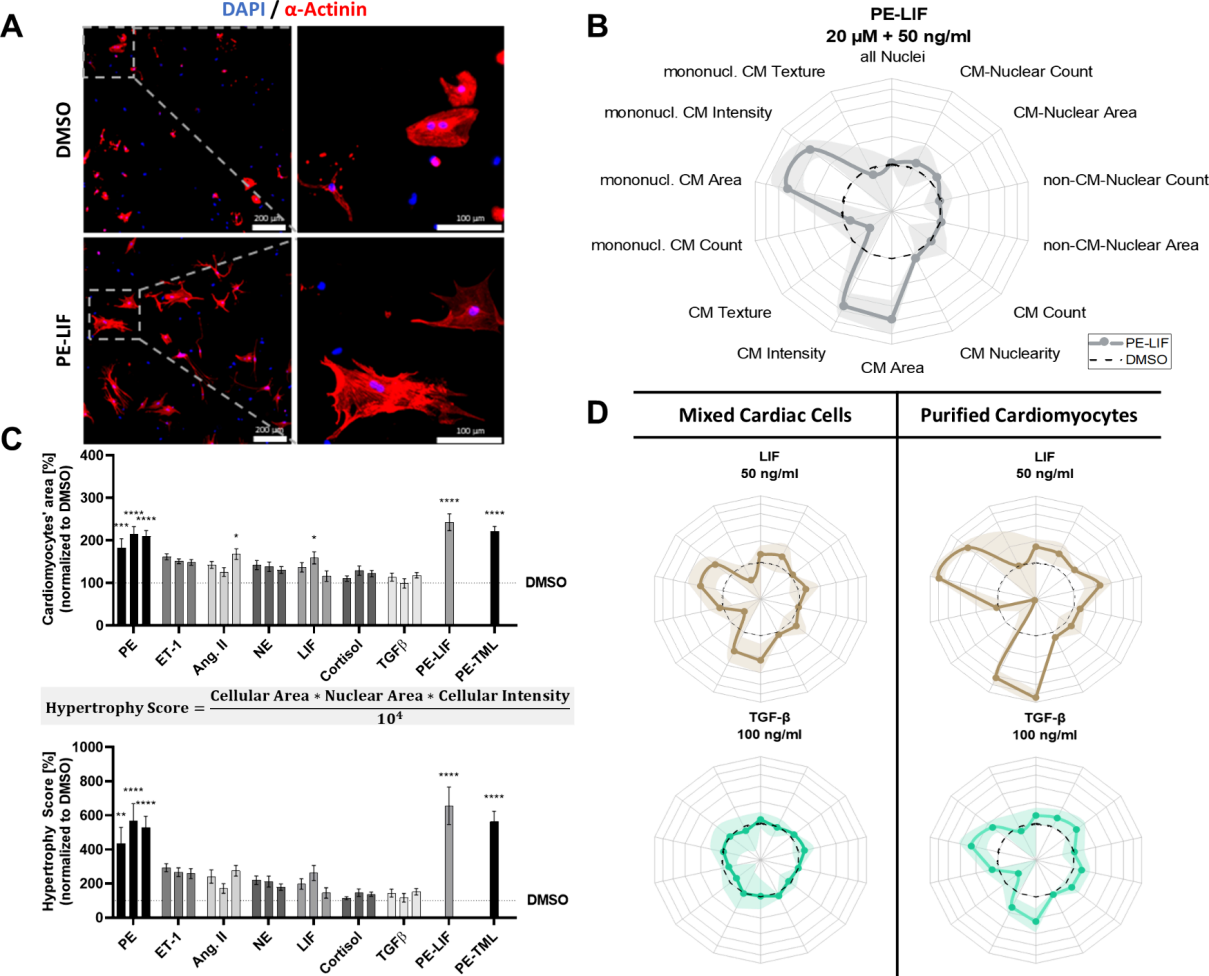
Systematic evaluation of cardiac hypertrophy stimulants defines distinct levels of phenotype induction (Hypertrophy Score) and morphometric signatures. (A) Representative immunofluorescence images of mixed cardiac cells treated with 20 μM PE and 50 ng/mL LIF or DMSO control. (B) Phenotypic profile of mixed cardiac cells following PE-LIF stimulation compared to DMSO control (*n* = 3, shaded area indicates SEM). (C) Hypertrophic stimulants were tested at three concentrations (left to right): phenylephrine (PE, 10/20/50 μM), endothelin-1 (ET-1, 50/100/200 nM), angiotensin II (Ang. II, 50/100/200 nM), norepinephrine (NE, 1/2/5 μM), leukemia inhibitory factor (LIF, 20/50/100 ng/mL), cortisol (1/2/5 μM), TGFβ-1 (50/100/200 ng/mL), PE-LIF (PE, 20 μM + LIF 50 ng/mL), PE-timolol (PE, 20 μM + TML, 2 μM). To facilitate direct comparison across conditions, key phenotypic parameters were integrated into a single metric, i.e., the Hypertrophy Score, which enhances the dynamic range of the assay (*n* = 3). (D) Cytokine-induced hypertrophy was more pronounced in purified CM cultures compared to mixed cardiac cells, highlighting model-specific differences in responsiveness (*n* = 3, shaded area indicates SEM). (Radar plot axes 50–350%, mean ± SEM, one-way ANOVA: * *p* ≤ 0.05, ** *p* ≤ 0.01, *** *p* ≤ 0.001, **** *p* ≤ 0.0001)

The three most consistently altered parameters (CM area, nuclear area, and α-actinin intensity) were combined into a single metric, the “Hypertrophy Score”, enabling direct quantitative comparison of hypertrophic stimuli and pharmacological interventions. All parameters were equally weighted in the calculation (Figure 2C). Comparing conventional CM area measurements with the composite Hypertrophy Score revealed similar trends but clearer distinctions between stimulant effects. Although both α- and β-adrenergic receptors are activated by catecholamines, their roles differ: β-receptors primarily regulate contractility, whereas α-receptors are more directly involved in hypertrophic growth.^26^ This was reflected in the Hypertrophy Score, where the α_1_-agonist PE induced the strongest response—whether alone or in combination with the β-antagonist TML or the cytokine LIF.^25^ Notably, PE consistently elevated the Hypertrophy Score across all conditions, with the PE+LIF combination producing the most pronounced hypertrophy, thus serving as the positive control. In contrast, NE, a nonselective adrenergic agonist, triggered a weaker response than PE alone. These results underline the dominant role of α-adrenergic signaling in promoting CM hypertrophy *in vitro*. ET-1 and Ang. II also demonstrated robust hypertrophic effects, reinforcing their known roles in cardiac remodeling.^22^

LIF, a cytokine of the IL-6 family, induces cardiac hypertrophy via activation of the gp130 receptor^23^ and downstream JAK/STAT and MAPK pathways,^27^ both central to hypertrophic remodeling.^28^ Additionally, LIF upregulates L-type Ca^2+^ channels, increasing calcium influx and activating Ca^2+^-dependent effectors like calcineurin and calmodulin, which promotes hypertrophic gene expression.^24^ By engaging multiple pro-hypertrophic pathways, LIF, especially in combination with adrenergic stimulation (PE), broadens and strengthens the dynamic range of the assay. Moreover, its activation of JAK/STAT signaling mirrors inflammatory processes,^29^ a hallmark of pathological hypertrophy, thereby enhancing the biological relevance of the *in vitro* model. As expected, LIF alone induced a strong hypertrophic response, which was further amplified when combined with PE.

To evaluate the advantages and limitations of the mixed cardiac cell approach, our assay was next directly compared to a protocol that enriches CMs through purification.^21^ Interestingly, the tested cytokines LIF and TGFβ had little to no effect in the mixed culture, whereas in purified CMs, LIF significantly increased the Hypertrophy Score, reaching a level comparable to PE stimulation (Figure 2D). This discrepancy may result from the absence of paracrine interactions in purified cultures, allowing stronger direct cytokine effects. These findings highlight the impact of model choice on experimental outcomes. To demonstrate the robustness of the assay across different imaging systems, we successfully validated its reproducibility using two independent microscopy platforms, with strong correlation of key hypertrophic features (Supporting Information, Figure S2). This confirms the applicability of the platform beyond a specific imaging setup, making it a versatile assay tool for the community.

## Target Validation and Profiling of a GRK5 Inhibitor Series

Given its high sensitivity to adrenergic hypertrophy induction, our assay should serve as a robust platform for drug discovery, particularly for *in cellulo* evaluation of compounds that modulate adrenergic signaling. A central regulator of this pathway is the G protein-coupled receptor (GPCR) kinase 5 (GRK5), which modulates adrenergic receptor activity^30^ and also translocates to the nucleus via a Ca^2+^/calmodulin-dependent mechanism in response to adrenergic stimulation.^31,32^ There, it influences hypertrophic gene transcription through NFκB^33^ and NFAT^34^ signaling and by phosphorylating HDACs, leading to MEF2 activation^31,32,35^ (Figure 3A). Targeting the noncanonical nuclear functions of GRK5 may enhance therapeutic precision while minimizing side effects. GRK5 dysregulation, especially under chronic adrenergic stress, contributes to pathological remodeling and heart failure.^6^ Due to high homology of GRKs with other AGC kinases, selectivity is critical to avoid off-target effects and preserve normal cardiac function.^36^

**Figure 3:**
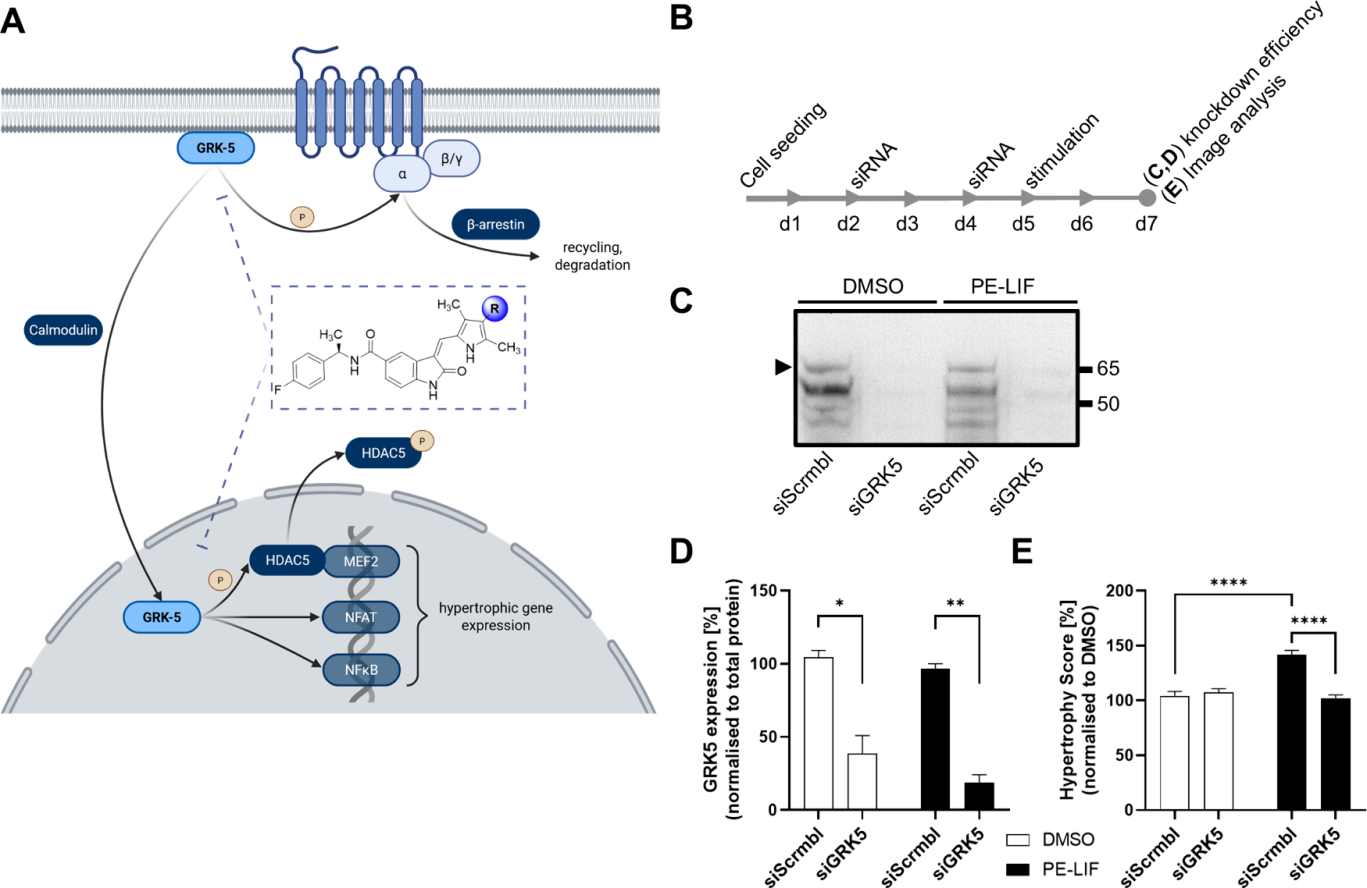
GRK5 is a tractable target in the new hypertrophy assay. (A) Role of GRK5 in the context of cardiac hypertrophy. The canonical pathway regulates GPCR signaling via phosphorylation of activated receptors, whereas the noncanonical pathway involves GRK5 translocation to the nucleus in a Ca^2^^+^/calmodulin-dependent manner where it regulates hypertrophic gene expression via phosphorylation of HDAC5 (created with biorender.com). (B–D) GRK5 siRNA knockdown in mixed cardiac cells (B) efficiently reduces protein levels as shown in representative immunoblots (C) and after semiquantitative blot analysis (D, *n* = 2), resulting in significant prevention of hypertrophy induction by PE-LIF (20 μM and 50 ng/mL) versus DMSO treatment (E, *n* = 3) (Mean ± SEM, unpaired *t* test; * *p* ≤ 0.05, ** *p* ≤ 0.01, *** *p* ≤ 0.001, **** *p* ≤ 0.0001).

Previously, GRK5 was validated as a tractable target only in purified cultures.^37^ To test if GRK5 is authentically represented in our new model, GRK5 expression was selectively reduced using siRNA and PE-LIF-induced hypertrophy was evaluated. A change in the experiment from 384-well to a 6-well format was necessary to obtain sufficient protein amounts for western blot analysis. Additionally, the cell density was increased to 750,000 cells per well in order to ensure adequate protein yield and to meet the siRNA transfection protocol requirements (Figure 3B). The results confirmed that the siRNA-mediated knockdown of GRK5 resulted in a reduction of its protein levels to 38% and 19% in DMSO and PE-LIF treated cells relative to total protein, as determined by quantitative immunoblotting (Figure 3C,D). Notably, despite switching from a 384-well to a 6-well format and the associated increase in cell density, the established CellProfiler pipeline remained fully applicable, although with a reduced dynamic range. Subsequent image analysis confirmed that PE-LIF stimulation increased the Hypertrophy Score to 141%, demonstrating robust detection of hypertrophy under these modified conditions (Figure 3E). Importantly, GRK5 knockdown successfully prevented this response, reducing the Score to baseline levels (102%), thereby validating GRK5 as a functional target in the mixed cardiac cell model.

One promising GRK5-selective inhibitor series derives from sunitinib,^38^ a 2-oxoindoline that exhibited GRK5 activity at an IC_50_ of 0.83 μM.^39^ Its optimization yielded compounds with nanomolar potency and improved selectivity for GRK5 over GRK2 by covalently targeting GRK5-specific Cys474.^39^ Combined SAR studies and X-ray structure analyses guided the introduction of reversible covalent warheads and even noncovalent analogs to balance potency and selectivity.^40^ A focused set of 2-oxoindoline derivatives (Figure 4A) with distinct GRK2/5/6 selectivity profiles and binding modes was selected to probe the herein developed high-content hypertrophy assay. Although extensive *in vitro* activity and SAR data exist for this inhibitor class, their cellular effects had not yet been reported. This study bridges that gap by assessing their functional activity in a disease-relevant cell-based model, offering new insights into their therapeutic potential in cardiac hypertrophy.

**Figure 4:**
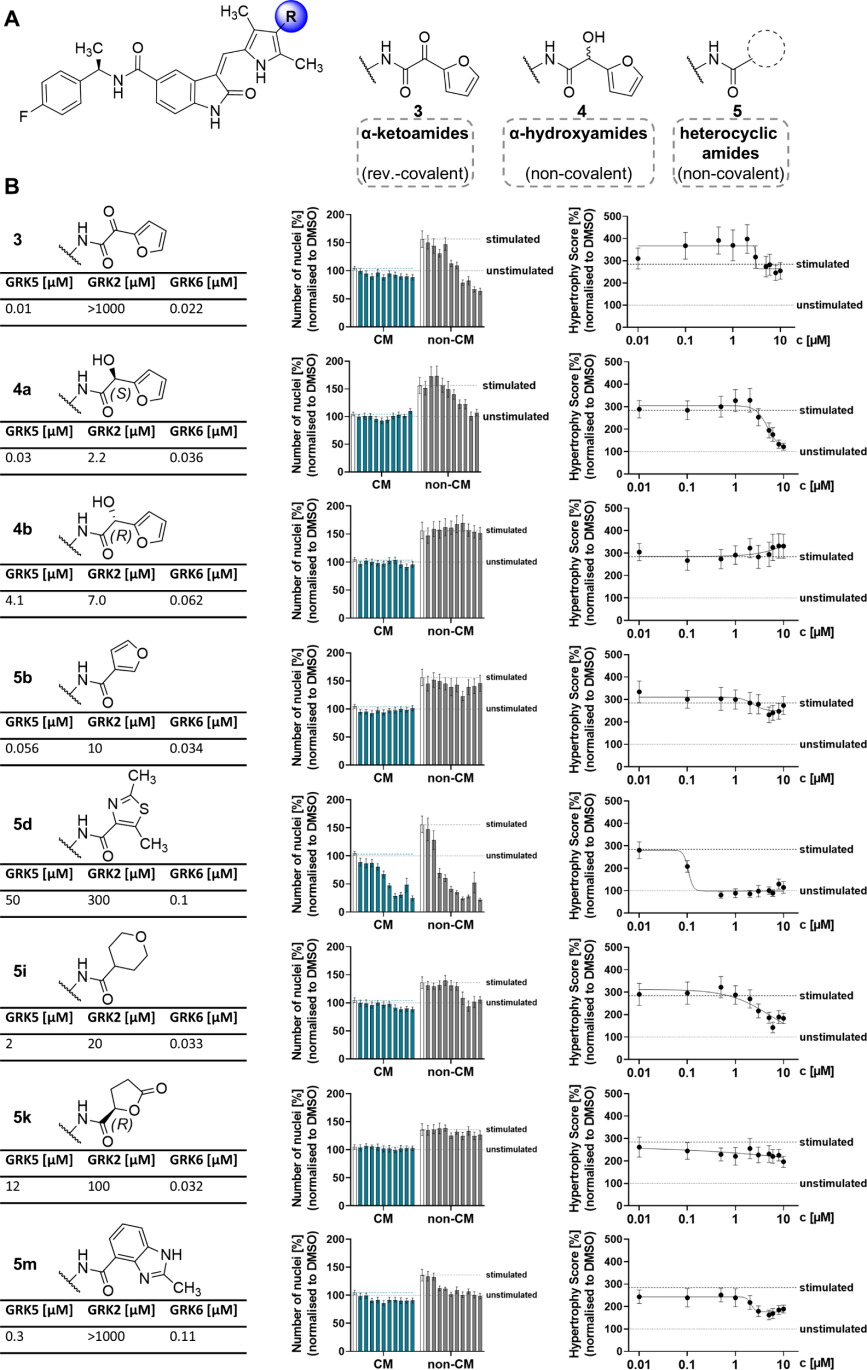
Phenotypic profiling of a sunitinib-derived GRK5 inhibitor series for *in cellulo* SAR as antihypertrophic agents. (A) Chemical structure of sunitinib-derived GRK5 inhibitors with reversible-covalent α-ketoamide warhead (**3**), α-hydroxyamide enantiomers (**4**) and a series of amides with distinct heterocyclic substituents (**5**). (B) Cardiotoxicity and antihypertrophic effects (Hypertrophy Score) were evaluated in mixed cardiac cells after 96 h stimulation with PE-LIF (20 μM, 50 ng/mL) and treatment with inhibitors (*n* = 3, mean ± SEM). Biochemical IC_50_ values are stated for each inhibitor.

To initially assess cardiotoxicity, nuclei counts of CMs and non-CMs were analyzed (Figure 4B). Inhibitor **5d** reduced non-CM numbers starting at 0.5 μM, with CMs affected at ≥2 μM. Given its low GRK5 potency (IC_50_ = 50 μM), these effects likely stem from off-target toxicity. This is critical for phenotypic interpretation, as stressed or dying cells may shrink and falsely mimic antihypertrophic responses. All other inhibitors did not exhibit obvious toxicity but did show reductions in non-CM numbers, which likely reflects antiproliferative activity. This effect was most pronounced for inhibitors **3** and **4a**, but absent for (*R,R*)-diastereomer **4b**, strongly suggesting that it is GRK5 inhibition related. Given the nonproliferative nature of postmitotic CMs, these reductions are most likely due to effects on proliferating cardiac fibroblasts. Cardiac fibroblasts are key contributors to pathological remodeling through proliferation and extracellular matrix deposition in response to stress. Their expansion is closely associated with the progression of cardiac hypertrophy.^11^ Therefore, the observation that GRK5 inhibitors may affect fibroblast proliferation, in addition to counteracting CM hypertrophy, might add a previously unrecognized facet to their therapeutic potential. Notably, this effect would not have been observed in conventional assays relying solely on purified CMs.

Evaluation of the Hypertrophy Score confirmed the cellular activity of the active (*R,S*)-diastereomer **4a** and the inactivity of its (*R,R*)-diastereomer **4b**, consistent *in vitro* data from radiometric kinase inhibition assays. Compound **4a** (GRK5 IC_50_ = 0.03 μM) significantly reduced the PE-LIF-induced Hypertrophy Score from 3 μM onward, whereas **4b** (GRK IC_50_ = 4.1 μM) showed no effect at any concentration. Interestingly, both **4a** (GRK6 IC_50_ = 0.036 μM) and **4b** (GRK6 IC_50_ = 0.062 μM) also inhibited ubiquitously expressed GRK6 in biochemical assays. Because **4b** showed no activity in the hypertrophy assay, the observed phenotype is likely not linked to GRK6 inhibition, consistent with its minor role in cardiovascular pathophysiology.^41^ Furthermore, although **4a** does exhibit weak activity against GRK2 (IC_50_ = 2.2 μM), this potency is insufficient to translate into significant cellular effects at the tested concentrations. In contrast, the strong activity of **4a** against GRK5 and the dose-dependent reduction in the Hypertrophy Score strongly suggest that the observed cellular effects are primarily due to selective GRK5 inhibition. However, no kinome profiles are currently available for **4a/b** to further substantiate GRK5-selectivity on a global kinase target scale within the herein probed disease context.

Inhibitor **5d** appeared to reduce hypertrophy but was excluded due to toxicity and low GRK5 potency. Compounds **5b** and **5k** showed neither toxicity nor significant antihypertrophic effects. In contrast, inhibitors **3**, **5i**, and **5m** partially reduced the Hypertrophy Score but did not restore it to DMSO baseline. As dose-dependence was inconsistent, the effect at 8 μM (I_8 μM_) was used for comparison (Figure 5A), revealing **4a** as the most potent cellular inhibitor, which was selected for orthogonal validation. To confirm cellular efficacy at the transcriptional level, a qPCR assay showed that **4a** prevented PE-LIF-induced upregulation of *Nppa* and *Nppb*, maintaining expression at DMSO control levels (Figure 5B). This supports the effectiveness of **4a** across biochemical, phenotypic and gene expression levels.

**Figure 5:**
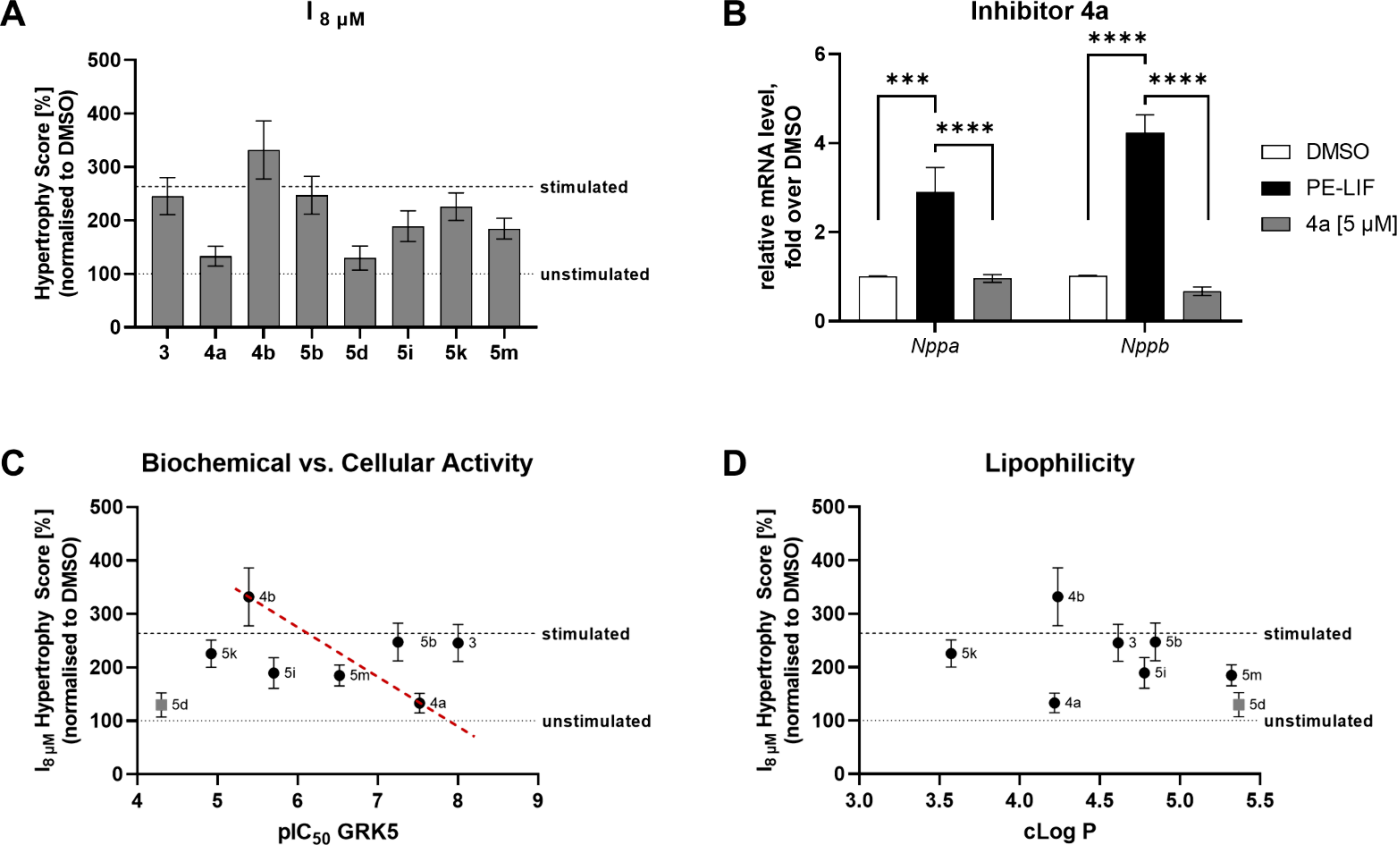
Integrated evaluation of antihypertrophic GRK5 inhibitor efficacies. (A, B) Side-by-side comparison of inhibitor efficacies at 8 μM (Hypertrophy Score) underline superior cellular activity of **4a** (A). Antihypertrophic efficacy was further validated by a significant reduction of hypertrophic gene expression (*Nppa* and *Nppb*) in purified CMs as determined by qPCR (B); data is shown as mean ± SEM (*n* = 4), one-way ANOVA: *** *p* ≤ 0.001, **** *p* ≤ 0.0001). (C) Correlation plot of biochemical GRK5 inhibition potency (pIC_50_) and antihypertrophic efficacy in the cellular assay (Hypertrophy Score, I_8 μM_). The dashed red line connects diastereomer pairs **4a/b** to visualize an optimal relationship of on-target potency and cellular efficacy, independent from physicochemical differences. Note: **5d** is marked in gray as an outlier due to likely cytotoxicity. (D) Relationship between lipophilicity (cLogP) and cellular efficacy (Hypertrophy Score, I_8 μM_); cLogP values were calculated using Schrödinger’s Maestro 14.3.129.

Despite similar biochemical potencies among most tested GRK5 inhibitors (except **5d**), their cellular efficacies varied substantially (Figure 5C). As expected, compounds with poor GRK5 potency (**4b**, **5k**) lacked cellular efficacy, whereas those with high potency (**4a**) showed clear cellular effects. Diastereomers **4a/b** present an excellent reference set due to (almost) identical physicochemical properties but opposing biological activities, resulting in a theoretically ideal correlation of biochemical and cellular potency (red-dotted line). However, inhibitors **3** and **5b** showed surprisingly poor cellular efficacy despite good GRK5 biochemical activity, prompting further analysis.

To identify possible explanations, the lipophilicity (cLogP) of each compound was calculated using the Schrödinger Maestro software, providing an initial assessment in the absence of experimental data (Figure 5D). Although lipophilicity can enhance passive permeability, excessive values may lead to membrane trapping and increased nonspecific binding due to hydrophobic interactions.^42^ Inhibitors **3** and **5b** displayed particularly high cLogP values, which likely contributed to their low cellular efficacy despite good GRK5 inhibition *in vitro*. In contrast, **4a** had a lower cLogP, suggesting that its superior performance in cells may be due to a better balance between lipophilicity and potency. In addition, the reversible-covalent α-ketoamide warhead in **3** might result in nonspecific binding events negatively affecting target engagement via unfavorable subcellular distribution.

Drug innovation in cardiology lags behind other fields, leaving a limited pipeline for new therapies.^43,44^ One reason is the complex pathophysiology that is difficult to mirror in relevant assay/model systems. Ideally, new targets, modes-of-action and modalities (early lead structures) undergo effective workflows already at early discovery stages for *in vivo* translation to demonstrate target/concept tractability. In this regard, meaningful *in vitro* assays based on rodent cells are vital, given that initial *in vivo* proof-of-concept studies are typically performed in rodent disease models.

We present an HTS hypertrophy assay that recapitulates the complex physiology of mixed rodent cardiac cell cultures, preserving intercellular and paracrine signaling. A custom CellProfiler pipeline enables high-content analysis, integrating multiple morphological features into a single ‘Hypertrophy Score’ for robust phenotypic comparison. The open-source workflow is widely applicable across different imaging systems and easily adaptable for future extensions. The platform enabled the very first reported cellular efficacy profiling of a small-molecular GRK5-selective inhibitor set. GRK5, a key regulator of maladaptive adrenergic signaling and pro-hypertrophic gene expression,^30^ was validated as a viable target in the new assay via siRNA knockdown, confirming its role in PE-LIF-induced hypertrophy.

From a chemotype-specific collection of GRK5 inhibitors,^40^ **4a** was characterized as the most promising candidate with strong antihypertrophic effects (cellular IC_50_ = 4.7 μM), further confirmed in an orthogonal biomarker assay (qPCR). Importantly, the optical antipode **4b**, which exhibited similar biochemical potency for GRK2 and GRK6 but no GRK5 inhibition, showed no antihypertrophic efficacy. These findings ruled out GRK2 and GRK6 as off-targets, suggesting that selective GRK5 inhibition is sufficient to counteract hypertrophy. Hence, **4a** and inactive **4b** present a valuable pair of chemical probes for advanced *in vivo* efficacy studies. Notably, several inhibitors (e.g., **4a**), also showed antiproliferative effects in non-CMs, indicating potential antifibrotic activity. Such effects would likely have been missed in conventional (purified) CMs assays, underscoring the biological relevance and translational advantage of the mixed-cell model.

By linking biochemical potency, lipophilicity and cellular efficacy, this study provides key insights and proof-of-concept for GRK5 as a therapeutic target. The assay delivers relevant cellular data to guide lead optimization, and is readily scalable for profiling larger compound collections from GRK5-centric medicinal chemistry campaigns. Hence, the assay platform is a powerful tool to accelerate *in vivo* validation of GRK5 and distinct inhibitor chemotypes. A clinically effective GRK5 inhibitor could offer a novel strategy to treat cardiac hypertrophy via modulation of tissue remodeling.

In the future, it will be interesting to leverage the introduced phenotypic assay system for emerging, underexplored targets that might be attractive for pharmacological cardiac hypertrophy management.

## Supplementary Material



## Notes

Detailed experimental procedures/methods and supplementary data including full characterization of hypertrophic stimulants and cross-platform imaging comparison for phenotypic evaluation (PDF)

## Notes

No unexpected or unusually high safety hazards were encountered.

## Abbreviations

Ang.II: angiotensin-II
ANP: atrial natriuretic peptide
BNP: B-type natriuretic peptide
CM: cardiomyocytes
CVDs: cardiovascular diseases
ET-1: endothelin-1
FBS: fetal bovine serum
GPCR: G protein-coupled receptor
GRK5: G protein-coupled receptor kinase 5
HDAC: histone deacetylases
LIF: leukemia inhibitory factor
MEF2: myocyte enhancer factor-2
NE: norepinephrine
NRCM: neonatal rat cardiomyocytes
P1: postnatal day 1
P5: postnatal day 5
PE: phenylephrine
TGFβ: transforming growth factor β
TML: timolol.
