# Supplementary material for: High-Throughput Cardiac Hypertrophy Phenotyping Supports Lead Optimization of GRK5 Inhibitors
Source: ACS Med Chem Lett. 2025 Dec 15;17(1):144–53. doi: 10.1021/acsmedchemlett.5c00528 (PMC12794055; doi:10.1021/acsmedchemlett.5c00528)
Supplement: Supplementary file 1 [file ml5c00528_si_001.pdf]

– Supporting Information –

High-throughput cardiac hypertrophy phenotyping supports lead optimization of GRK5 inhibitors

Pia Steinkuhl,[1,2,3] Anca Kliesow Remes,[2,3] Carmen Carrillo García,[1] Amol Sonawane,[4] Ranjith Kumar Gadi,[4] Arun K. Ghosh,[4,5] John J G Tesmer,[5,6] Oliver J Müller,[2,3] Dennis Schade[1,3]*

[1] Department of Pharmaceutical & Medicinal Chemistry, Christian-Albrechts-University of Kiel, 24118 Kiel, Germany

[2] Department of Internal Medicine V, University Medical Center Schleswig-Holstein, Campus Kiel, 24105 Kiel, Germany

[3] German Center for Cardiovascular Research (DZHK), partner site Hamburg/Kiel/Lübeck, 24105 Kiel, Germany

[4] Department of Chemistry, Purdue University, West Lafayette, IN 47907, USA

[5] Department of Medicinal Chemistry and Molecular Pharmacology, Purdue University, West Lafayette, IN 47907, USA

[6] Department of Biological Sciences, Purdue University, West Lafayette, IN 47907, USA

* Lead contact: schade@pharmazie.uni-kiel.de

Table of Contents

Supplemental Tables and Figures 2

Table S1 2

Figure S1 3

Figure S2 4

Experimentals 6

Animal experiments 6

Isolation of primary cells 6

siRNA Knockdown 6

Protein Analysis 6

qPCR 7

High-Content Imaging 7

Statistical Analysis 8

Supplemental References 10

Supplemental Tables and Figures

Table S1

Table S1: Overview of tested hypertrophic stimulants.

Stimulants	Compound Structure	Target	Concentrations
Phenylephrine (PE)	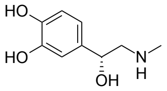	Alpha-1 adrenergic agonist (GPCR)	10; 20; 50 µM
Norepinephrine (NE)	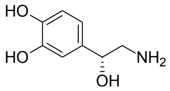	Alpha- and beta-adrenergic agonist (GPCR)	1; 2; 5 µM
Timolol (TML)	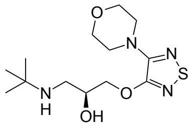	Beta-adrenergic antagonist (GPCR)	2 µM (+ PE 20 µM)
Cortisol	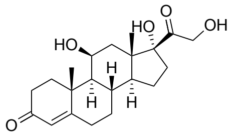	Cortisol receptor (nuclear receptor, transcription factor)	1; 2; 5 µM
Endothelin-1 (ET-1)	Cys-Ser-Cys-Ser-Ser-Leu-Met-Asp-Lys-Glu-Cys-Val-Tyr-Phe-Cys-His-Leu-Asp-Ile-Ile-Trp	Endothelin-1 receptor (GPCR)	50; 100; 200 nM
Angiotensin II (Ang. II)	Asp-Arg-Val-Tyr-Ile-His-Pro-Phe	Angiotensin II receptor (GPCR)	50; 100; 200 nM
Leukemia inhibitor factor (LIF)	[cytokine of IL-6 family]	Gp130 receptor associated to LIF receptor	20; 50; 100 ng/ml
TGFβ-1	[cytokine of TGFβ family]	TGFβ receptor (transmembrane serine/threonine kinase)	50; 100; 200 ng/ml

Figure S1

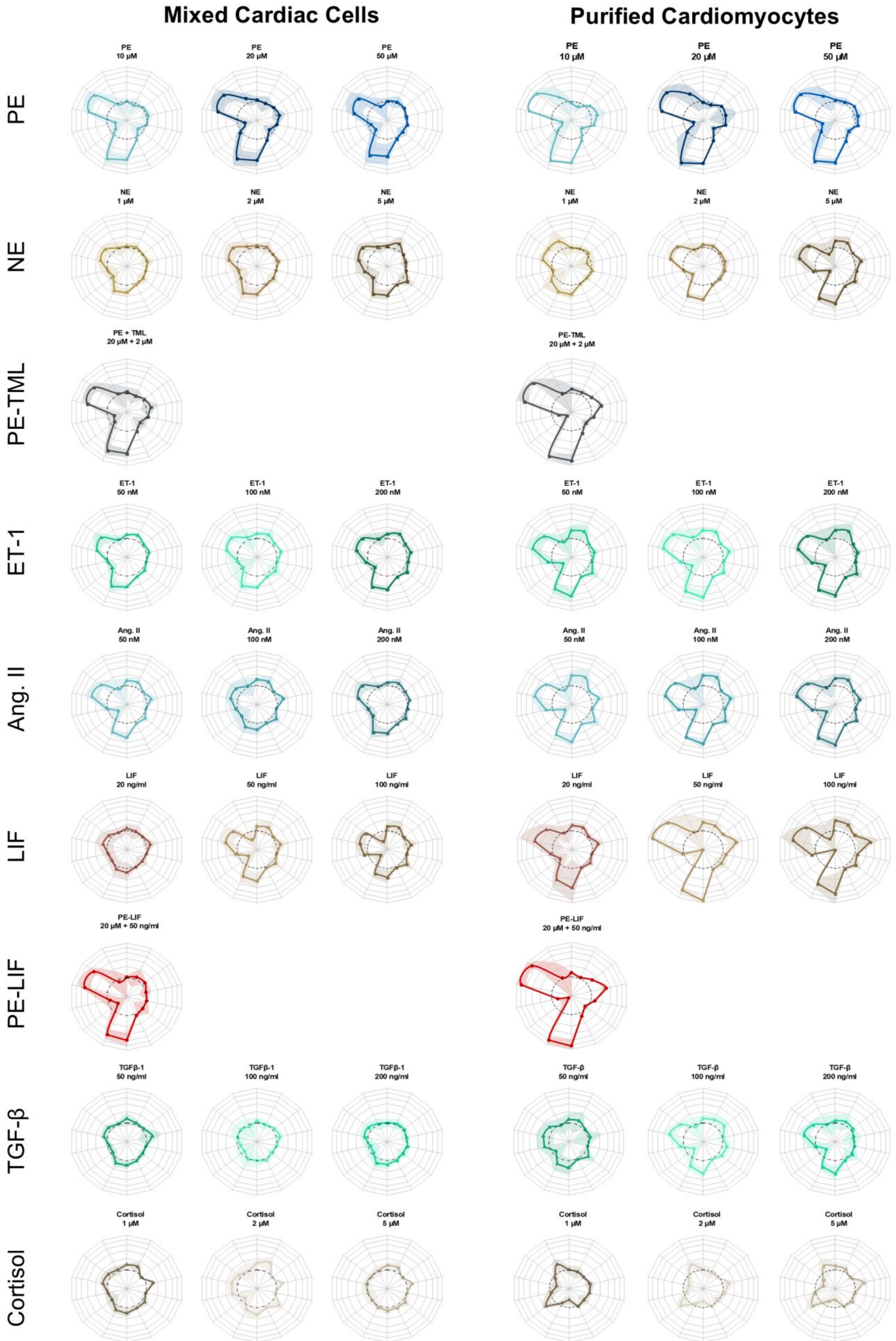

Figure S1: Overview of hypertrophy phenotypes induced by distinct stimuli. Phenotypic profiles of hypertrophic stimulants (phenylephrine (PE), norepinephrine (NE), timolol (TML), endothelin-1 (ET-1), angiotensin-II (Ang. II), leukemia inhibitory factor (LIF), transforming growth factor β1 (TGFβ-1), and cortisol) are visualized as radar plots. Fingerprint analyses are shown for all tested concentrations in both mixed and purified cardiomyocyte cultures. (n = 3, normalized to DMSO, axis range from 50-350%, mean ± SEM)

Figure S2

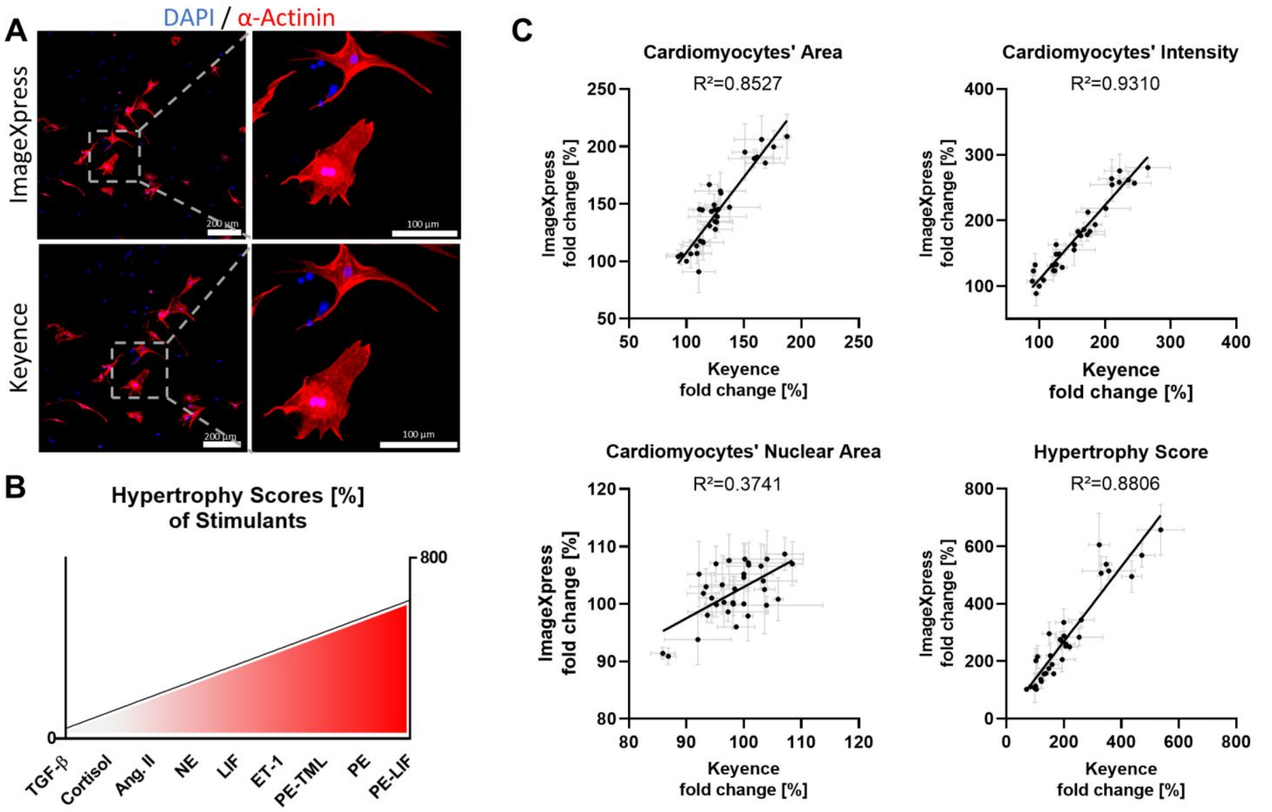

Figure S2 Cross-platform comparison of hypertrophic phenotype analysis using two different imaging systems. (A) Representative immunofluorescence images of PE-LIF (20 µM and 50 ng/mL) stimulated cells were acquired using the ImageXpress XL (Molecular Devices) and BZ-X800 (Keyence) imaging platforms. **(B)** Overview of Hypertrophy Scores from hypertrophic stimulant screening, which were used in the same order for correlation analyses shown in (C). **(C)** Comparison of hypertrophic metrics show strong correlations between imaging instruments for cardiomyocyte area and intensity, whereas nuclear area correlates less robustly, likely due to sensitivity in nuclear segmentation. The Hypertrophy Score remained consistent between systems. (n = 2, mean ± SEM)

Because image quality is an essential part of phenotypic analysis, we assessed whether this assay is robust across two different imaging systems. Two representative plates from the hypertrophic stimulant experiment were analyzed using two different microscopy platforms: the ImageXpress (the standard system for this study) and the Keyence BZ-X810 Fluorescence Microscope. Although the Keyence microscope was not equipped with a high-throughput tool, its higher image quality and capability to capture z-stacks and stitched images offer advantages in certain settings. Instead of capturing multiple individual sites per well, the Keyence stitched together four images per well, resulting in fewer images overall and a faster analysis pipeline.

Both imaging systems exhibited strong correlations for hypertrophic parameters (Figure S2). Cardiomyocyte area and intensity measurements correlated well. However, the correlation for nuclear area was unexpectedly lower. This may be due to the fact that nuclei are relatively small objects, making their segmentation more sensitive to slight differences in image resolution, magnification or stitching. Even minor variations in how nuclear boundaries are detected can significantly affect area measurements. In contrast, larger structures like cardiomyocytes are less

affected by these subtle differences, resulting in more consistent measurements across imaging systems. Despite this, the Hypertrophy Score itself maintained a strong correlation ($R^2 = 0.8806$), confirming that both imaging systems can effectively characterize hypertrophic phenotypes.

Experimentals

Animal experiments

The animal studies were approved by the relevant authorities of Schleswig-Holstein, Germany (internal permit no 1085), and procedures were performed in accordance with the guidelines from Directive 2010/63/EU of the European Parliament on the protection of animals used for scientific purposes.

Isolation of primary cells

Mixed cardiac cells were isolated from neonatal rat hearts as previously described [86]. Briefly, five-day-old (P5) Wistar rats were sacrificed by decapitation. The heart was harvested and placed in sterile PBS buffer on ice. Residual tissue or blood was removed with tweezers under the microscope. Subsequently, enzymatic and mechanical digestion of the organ was performed using Miltenyi's Neonatal Heart Dissociation Kit as described in the manufacturer's protocol. Cells were centrifuged at 55 *g* for 15 min and the supernatant was aspirated and replaced with DMEM (DMEM, 2% FBS, 1% penicillin/streptomycin).

To purify cardiomyocytes, P1-3 Wistar rats were sacrificed by decapitation, and hearts were harvested and placed in sterile buffer A. Dissociation was done mechanically by scissors and using a mix of pancreatin and collagenase type II in three cycles for 20 min at 37 °C while shaking. After each cycle, the dissociation solution was replaced and the isolated cells were pooled, strained with a 100 µm cell strainer and 10 ml FBS was added to halt the dissociation. Cells were centrifuged at 220 *g* for 5 min and the pellet was resuspended in buffer A. To purify cardiomyocytes from other cardiac cell types, Percoll gradient centrifugation was performed as described [1].

Cells were seeded on gelatin (0.1%) coated plates, either 1,500 cells per well in black 384-well plates (Greiner µclear) or 1,000,000 cells per well in 6-well plates.

Compound treatment and hypertrophy stimulation were added one day after seeding, typically using a combination of phenylephrine (PE) at 20 µM and leukemia inhibitory factor (LIF) at 50 ng/ml. For qPCR, cells were stimulated with 100 µM PE along with 50 ng/ml LIF.

siRNA Knockdown

The knockdown protocol was adapted from a previously published method [2], with modifications according to the manufacturer's protocol of Lipofectamine RNAiMAX Reagent (Invitrogen). In 6-well plates, knockdown was performed twice, 48 h and 96 h after seeding with a final siRNA amount of 25 pmol. One day after the last knockdown, mixed cardiac cells were treated with either PE-LIF or DMSO dissolved in DMEM, in this step P/S was omitted to reduce stress on the cells. 48 h later, cells were fixed for immunocytochemistry or total protein was extracted to confirm knockdown efficiency via western blotting.

Protein Analysis

Media was aspirated and mixed cardiac cells were washed twice with warm sterile PBS. Then, trypsin was added to each well and incubated for 5 min at 37 °C. To detach remaining adherent cells, plates were gently tapped. To stop the activity of trypsin, 700 µl DMEM containing 2% FBS were added and mixed thoroughly. The content of each well was centrifuged in a 1.5 ml microtube for 3 min at 200 *g* at 4 °C. The supernatant was quickly aspirated and the cell pellet was resuspended in 30 µl of lysis buffer (10 mM 4-(2-hydroxyethyl)-1-piperazineethanesulfonic acid (HEPES), 10 mM KCl, 0.1 mM ethylenediaminetetraacetic acid (EDTA), 1 mM dithiothreitol, 0.5% Nonidet-P40, pH 7.5, phosphatase inhibitor cocktail and complete protease inhibitor cocktail). During 20 min of incubation on ice, the suspension was mixed by pipetting up and down. The final centrifugation step was performed for 10 min at 4 °C and 12,000 *g*.

The total amount of protein was quantified using Thermo Scientific's Pierce Bicinchoninic Acid (BCA) Protein Assay. Protein expression was evaluated by SDS-PAGE with a 12% self-cast gel, followed by western blotting on a PVDF membrane, according to standard procedures (150 µg protein loading). For normalization, 0.5% of 2,2,2-trichloroethanol (TCE) was incorporated in all separation gels for fluorescent visualization. GRK5 was stained with antibody from Santa Cruz (mouse monoclonal, Sc-518005, 1:500) and secondary HRP-conjugated antibody from Proteintech (goat anti-rabbit, SA00001-1, 1:1,000). The ECL-signal was imaged using a ChemoStar Touch ECL & Fluorescence Imager. Quantification of lane intensity was performed using ImageJ and the ECL signals of specific proteins were normalized to total protein from the TCE signal.

qPCR

RNA was extracted from cells cultured in 6-well plates after 24 h of treatment. Cells were washed twice with warm PBS, lysed and RNA was extracted using RNeasy Mini Kit with the RNase-Free DNase Set (Qiagen), following the manufacturer's instructions. RNA concentration was measured using the NanoDrop One^{C} spectrophotometer. Complementary DNA (cDNA) synthesis was done using the qScript cDNA Synthesis Kit. The qPCR reaction mix was prepared with a total volume of 15 µl per sample. This included 7.5 µl of Takyon SYBR Master Mix, 1.5 µl of forward and reverse primer mix (final concentration of 500 nM) and 0.75 µl of cDNA diluted with 5.25 µl RNase-free water. The qPCR was performed on the Bio-Rad CFX Opus 96 thermocycler. Gene expression was calculated using the $\Delta\Delta$Cq method [3] with *Rpl32* used as a housekeeping gene. Unstimulated DMSO-treated samples served as the control group for normalization.

High-Content Imaging

For 384-well plates, the media was aspirated and washed automatically using a BioTek 405 TS Plate Washer, while 6-well plates were handled manually. First, cells were fixed for 10 min with 4% formaldehyde in PBS. Subsequently, plates were washed thrice with PBS. Cells were then permeabilized using 0.2% TritonX and blocked using 5% FBS in sterile PBS (staining buffer, SB) for 20 min at room temperature. SB was aspirated and replaced by primary antibody for α-actinin (mouse monoclonal, Sigma A78119, 1:800) diluted in SB for 1.5 h at room temperature. Primary

antibody was washed off three times with PBS before addition of secondary antibody (goat anti-mouse, Alexa Fluor 568, Invitrogen A11004, 1:1,000) and DAPI (Roth), both diluted in PBS, were added for 1 h at room temperature in the dark. Finally, plates were washed three times with PBS before imaging with the ImageXpress Micro XL Widefield High Content Screening System (Molecular Devices). Capture settings consisted of a 10x Plan Fluor objective, 25 °C and laser auto-focus. Each well was imaged at 4 sites in both DAPI and Texas red channel. For higher resolution and magnification, the Keyence B7-X810 Fluorescence Microscope was used with a Plan Apo 10x objective. Similarly, four sites per well in both channels were captured. However, a z-stack was compiled from seven images to enhance image quality.

A semi-automated image analysis pipeline was established using CellProfiler (Broad Institute), an open-source software that enables modular, reproducible high-throughput analysis independent of specific hardware or computational infrastructure.

First, Illumination correction was applied to α-actinin images to address uneven lighting and improve signal-to-noise ratio, thereby enhancing segmentation accuracy. Segmentation for nuclei and cardiomyocytes was performed individually, taking into account the tendency of cardiomyocytes to mature to a binucleated state. Nuclei with homogenous size and morphology were segmented using the “Otsu two-class method”, categorizing pixels into a foreground and a background category based on an automated threshold calculation. To accurately identify clumped nuclei, the size range (14-17 px) was set narrowly and out of range nuclei were filtered out in a subsequent module. For the more heterogeneous morphology of cardiomyocytes, segmentation was done by the “robust background” method, allowing a better adjustment to varying image quality and identification of small cytoplasmic projections. Similarly to nuclear identification, a narrow size range (40-200 px) was applied for segmentation with subsequent filtering of out of range cardiomyocytes. Next, cardiomyocyte and non-cardiomyocyte nuclei were distinguished by co-localizing DAPI and α-actinin signals, to enable a more precise evaluation of the heterogeneous cell culture model. For this, the "MaskObjects" module was applied three times overlaying segmented nuclei and cardiomyocytes (CM) for CM-nuclei, non-CM-nuclei and cardiomyocytes (eliminating incorrectly identified cells, cell debris or cytoplasmic projections without nuclei). Because cardiomyocytes frequently exhibit binucleation as part of their growth process, nuclearity (the number of nuclei per cardiomyocyte) was included as a parameter. The “RelateObjects” module was used to assign all nuclei within a single cardiomyocyte as child objects of that cell. This also enabled the creation of subpopulations, such as filtering for mononucleated cells. For all nuclear and cellular subpopulation, various morphological measurements were calculated including quantitative measurements, nuclear and cellular area, cellular intensity and texture.

Statistical Analysis

All statistical analyses were calculated using GraphPad Prism 8.4.2 software. Comparisons of three or more groups were assessed using one-way ANOVA, followed by Dunnett’s multiple comparisons

test for specific pairs of groups. An unpaired t-test was used for comparison of two groups. p-values ≤ 0.05 were considered statistically significant (* $p \leq 0.05$, ** $p \leq 0.01$, *** $p \leq 0.001$, **** $p \leq 0.0001$). All experiments were conducted in experimental triplicate, each consisting of plate triplicates and technical triplicates on each plate. Data are presented as mean ± SEM.

Supplemental References

[1] Remes, A.; Wagner, A. H.; Schmiedel, N.; Heckmann, M.; Ruf, T.; Ding, L.; Jungmann, A.; Senger, F.; Katus, H. A.; Ullrich, N. D.; Frey, N.; Hecker, M.; Müller, O. J.: AAV-mediated expression of NFAT decoy oligonucleotides protects from cardiac hypertrophy and heart failure; *Basic Res Cardiol*. **2021** *116* (1) 38.

[2] Schlegel, P.; Reinkober, J.; Meinhardt, E.; Tscheschner, H.; Gao, E.; Schumacher, S. M.; Yuan, A.; Backs, J.; Most, P.; Wieland, T.; Koch, W. J.; Katus, H. A.; Raake, P. W.: G protein-coupled receptor kinase 2 promotes cardiac hypertrophy; *PLoS One*. **2017** *12* (7) e0182110.

[3] Pfaffl, M. W.: A new mathematical model for relative quantification in real-time RT-PCR; *Nucleic Acids Res*. **2001** *29* (9) e45.
